# Shall the Majority Rule?

**Published:** 1887-04

**Authors:** 


### EDITORIAL DEPARTMENT.

F. E. DANIEL, M. D., EDITOR AND PUBLISHER.

COLLABORATORS:

*E. J. Doering, M. D., Chicago.*
*Geo. Cupples, M. D., San Antonio.*
*Odo Betz, M. D., Germany.*
*E. J. Beall, M. D., Fort Worth, Texas.*
*T. C. Osborn, M. D., Cleburne, Texas.*
*Wm. Penny, M. D., New York.*
*H. O. Marcy, M. D., Boston.*
*C. K. Gregg, M. D., Mexico.*
*R. M. Swearingen. M. D., Austin.*
*C. H. Wilkinson, M. D., Galveston.*
*J. W. McLaughlin, M. D., Austin.*
*R. H. L. Bibb, M. D., Mexico.*

### SHALL THE MAJORITY RULE?

### A NEEDED AMENDMENT TO OUR CONSTITUTION.

In all republican governments the will of the majority must rule; in politics, indeed in conventions, and committees, and mass meetings and assemblies of all kind, whether political, business, ecclesiastical or what not, the observance of this custom has made it the law of the land; and it is provided by statutes.

The Texas State Medical Association is a singular exception to this rule, in its practical workings; notwithstanding it seems to have been the aim of the framers of the constitution to conform to the established custom. It is very evident that the principle of justice is recognized, that representation shall be on a basis of population (membership)—yet as said, in the practical workings of our conventions, the *minority* elect all officers, and decide all questions before the nominating committee!

The Constitution of the Texas State Medical Association, reads, (Article VII):

"County Medical Associations shall be entitled to two delegates to the meetings of this association, for every ten members comprising their societies, and one for every additional five members, *and shall have two votes upon all questions*; (italics ours.—Ed.) and all medical colleges in the State, holding charters from the State, with seven professors, shall be entitled to two votes; and no member shall be allowed to vote who is not a delegate of some association or medical college, except such member live in a county where no association exists, said county delegates being entitled to one vote."

A strict construction of this Article would give each County Medical Association *two votes*—no more, no less—without regard to the membership; and would cut off the right of a member to vote unless he be a delegate from some local association, or some college, or come from a county where there is no organization. The meaning being, at least, obscure, the question was referred to the Judicial Council for construction. The Council reported as follows, and their report is incorporated into the Constitution in the published volumes of the Association:

"The Judicial Council interprets the meaning of Article VII of the Constitution, as follows: Each member of the Texas State Medical Association who is in good standing, shall be entitled to one vote, and if he be also a delegate from a medical society or college properly authorized, shall be entitled to an additional vote. County Medical Associations shall be entitled to send two delegates for the first ten members, and one for each additional five members." [That is to say, one representative of every five members or fraction of five, after the first ten, in each county medical organization.]

From the above, it is very clear that it is intended that representation shall be on a basis of numbers, (membership.) But, the memory of the oldest member runneth not back to the time when any delegate on the floor of any meeting of this Association, ever cast any other than his individual vote by right of his membership, on any question that ever came up in general session, while in the Nominating Committee, which corresponds to nominating conventions in politics, and where every physician affiliated with the State Association by membership in a local society *should be represented*, only one representative of each county (and whether there be in that county an organization or not) is entitled to vote; and he casts *only one*—his individual vote! Was there ever such inconsistency? Practically, the delegate from a remote and sparsely settled county which boasts perhaps, not a half dozen physicians, and perhaps only this one member of the State Association, wields as much influence, and has as much voice in the election of officers, as the representative of the most populous county, having the largest and most active medical organization, with its ten or more delegates on the ground! Where is the necessity of sending "one delegate for every five members"? Only one goes into the nominating committee, and he casts, only one vote! Travis county, for instance, has an active medical organization with a membership of 31. This association sends up seven delegates. These seven delegates—all being members of the State Association (we will suppose) are clearly entitled to fourteen votes on the floor of the general sessions; and in choosing a spokesman for the nominating committee, they are supposed to delegate their votes to him; and by every principle of right and justice, law, custom and usage, this spokesman should cast seven votes on all questions that may arise; yet he casts one; hence Travis County Medical Society is not represented, and has no more voice than a county with a single member and no local organization!

Under these circumstances it seems meaningless to instruct delegates.

The above acquires additional force from the fact that each county association is taxed 50 cents *per capita* of its membership. In this, the fundamental principles of the American Government are violated, for the Constitution declares that "there shall be *no* taxation without representation." For what do these members of local societies pay? Many of them are not members of the State Association.

So long as we continue to work as we have been doing in this matter, it will be useless to urge "organization"—and the JOURNAL'S "occupation" will be "gone"; for, practically, the object of organizing local societies is defeated; there is no encouragement to labor to bring the entire regular profession within the fold, *if* they cannot be heard in council.

As any proposal to amend the Constitution of the State Association must lie over one year, we write the above to call attention of members to the necessity of some such action at this meeting; and it is hoped that some one who has the good of the cause at heart, will act on the suggestion; will introduce a resolution to so amend the Constitution as to secure the ends clearly had in view in framing it, and which are dictated by every consideration of right and custom; delegates should cast the votes of their constituency, as is done in every other convention of whatever kind, in all republican governments on the face of the earth.

